# Hardness data related to pre-ageing, natural secondary ageing, and paint bake hardening in Al-Mg-Si alloys

**DOI:** 10.1016/j.dib.2019.104494

**Published:** 2019-09-16

**Authors:** Zi Yang, Zeqin Liang, David Leyvraz, John Banhart

**Affiliations:** a Helmholtz Centre Berlin, Hahn-Meitner-Platz 1, 14109, Berlin, Germany; b Novelis R&T Centre, Sierre, Rte des Laminoirs 15, 3960, Sierre, Switzerland

**Keywords:** Al–Mg–Si alloys, Age-hardening, Pre-ageing

## Abstract

The effect of pre-ageing (PA) time and temperature on subsequent natural secondary ageing (NSA) and paint bake (PB) hardening of an AA6014 Al–Mg–Si alloy was systematically investigated, especially when both parameters change. A wide range of PA conditions was covered with temperatures ranging from 80 °C to 160 °C and times from several minutes to several days depending on the PA temperature. Hardness data for such pre-treatments measured by Brinell method are given. Hardness data measured during NSA are fitted by various functions. This dataset might be reused for further kinetic analysis of the clustering in Al–Mg–Si alloys or for the determination of the optimal PA tactics for industrial production.

**Table:** Specifications Table

Subject	*Materials Science* *Metals and Alloys*
Specific subject area	Clustering and precipitation in Al–Mg–Si alloys
Type of data	Table Figure
How data were acquired	Hardness testing Equipment: Qness model 60 M, Qpix Control software
Data format	Raw Analysed
Parameters for data collection	Room temperature, Brinell method, 10 kgf loading, 1 mm diameter tungsten carbide indenter, 10 s holding time, 8 indentations per sample.
Description of data collection	Diameter of the indentation was measured. Hardness value was calculated using Brinell method. The evaluation was performed with the help of the software Qpix Control.
Data source location	Helmholtz-Centre Berlin, Hahn-Meitner-Platz 1, 14109 Berlin, Germany
Data accessibility	Repository name: Mendeley Data DOI: 10.17632/zp69dm526p.1
Related research article	Author's name: Zi Yang, Zeqin Liang, David Leyvraz, John Banhart Title: Effect of pre-ageing on natural secondary ageing and paint bake hardening in Al–Mg–Si alloys Journal: Materialia DOI: https://doi.org/10.1016/j.mtla.2019.100413

**Table:** 

**Value of the data** • Why are these data useful? This dataset contains a systematic evaluation of the hardness after various pre-ageing (PA), natural secondary ageing (NSA), and paint bake (PB) hardening. PA conditions covered a wide range of time and temperature combinations, which enables a comprehensive understanding of the effect of PA on the subsequent NSA and PB. • Who can benefit from these data? Researchers who investigate the kinetics of clustering and precipitation in Al–Mg–Si alloys as well as the material manufacturers for the optimisation of the PA process in industrial production can benefit from the data. • How can these data be used for further insights and development of experiments? Fig. 5 shows an example how these data can be used to determine the best PA tactic for the material manufacturer. By overlaying the hardness plots before PB over after PB after certain NSA time, one can go for the area (corresponding to the PA condition) where the NSA hardness is within the limitation while the PB hardness is the highest. • What is the additional value of these data? The dataset might also be useful for the validation of the future multi-stage ageing modelling in Al–Mg–Si alloys.

## 1 Experimental design, materials, and methods

**Fig. 1:**
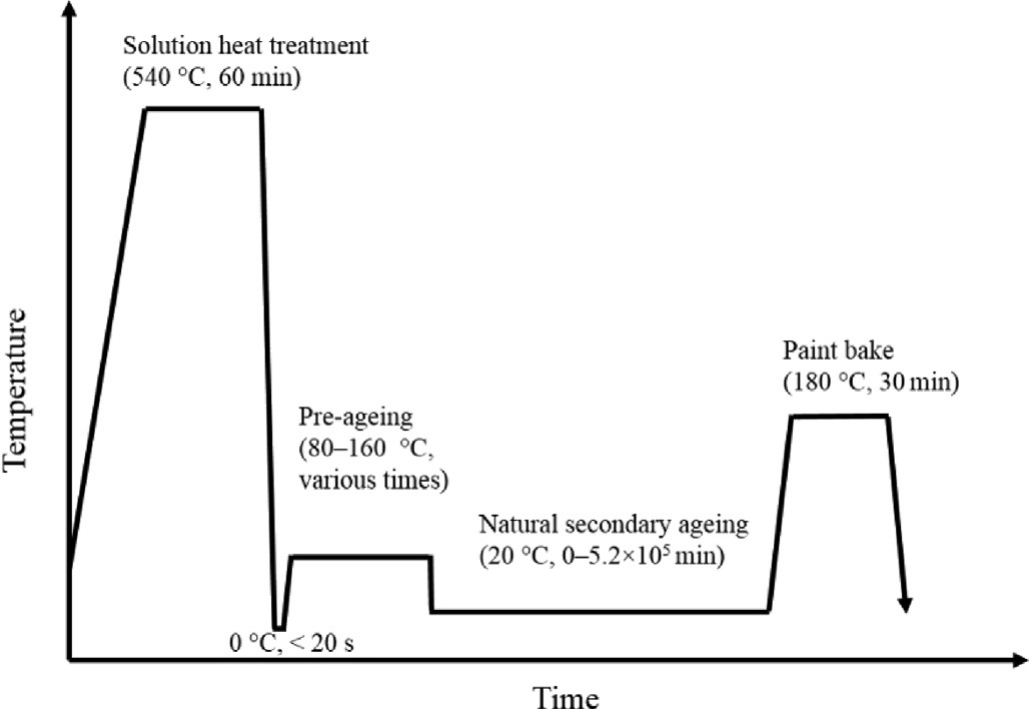
Schematic heat treatment program of multi-stage ageing processes.

Commercial alloy AA6014 (0.65% Mg, 0.60% Si, 0.18% Fe, 0.08% Mn, 0.12% Cu, all by mass) was provided by Novelis Switzerland. Solutionising (SHT) was performed in an air circulation furnace at 540 °C for 1 h. After quenching into ice water samples were quickly dried and immersed in an oil bath for PA to avoid unnecessary natural ageing (NA) or stored in a Peltier-cooled incubator for intended NA. PA temperatures and times are given in Table 1, Table 2. Artificial ageing (AA) at 180 °C for 30 min was applied to simulate an industrial paint-bake (PB) process. The entire heat treatment is defined by Fig. 1. Brinell hardness tests were performed on 10 × 10 × 1 mm^3^ large samples with a Qness Company model 60 M hardness tester using 10 kg loading force, a 1 mm diameter tungsten carbide indenter and holding for 10 s for each of the 8 indentations.

**Table 1:** Abbreviations used and definitions of heat treatment steps.

Code	Heat treatment	Conditions
SHT	Solution heat treatment	1 h at 540 ± 2 °C
NA	Natural ageing	20 ± 0.1 °C
NSA *z*	Natural secondary ageing for *z* min	20 ± 0.1 °C
PA *x/y*	Pre-ageing at *x* °C for *y* min	80 °C–160 °C, ± 0.2 °C
PB	Paint-bake	30 min at 180 °C, ± 0.2 °C

**Table 2:** PA conditions for NSA and PB response measurements.

Temperature (°C)	Time (min)
80	30, 240, 480, 1440, 2400, 4320, 5760
100	10, 60, 240, 480, 960
120	10, 30, 60, 120, 180, 240, 360
140	10, 30, 45, 60, 90
160	5, 10, 15, 20, 30

## 2 Data

**Fig. 2:**
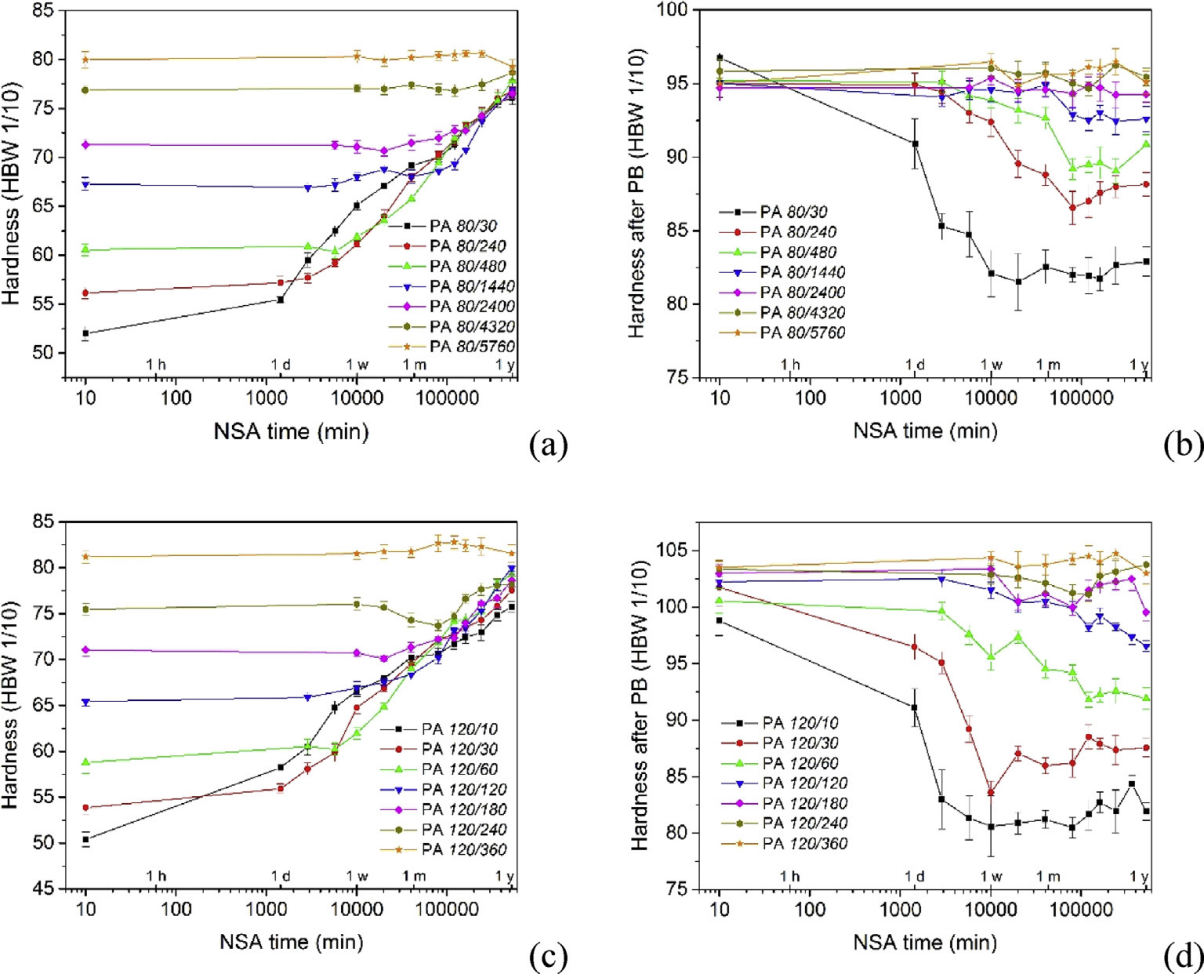

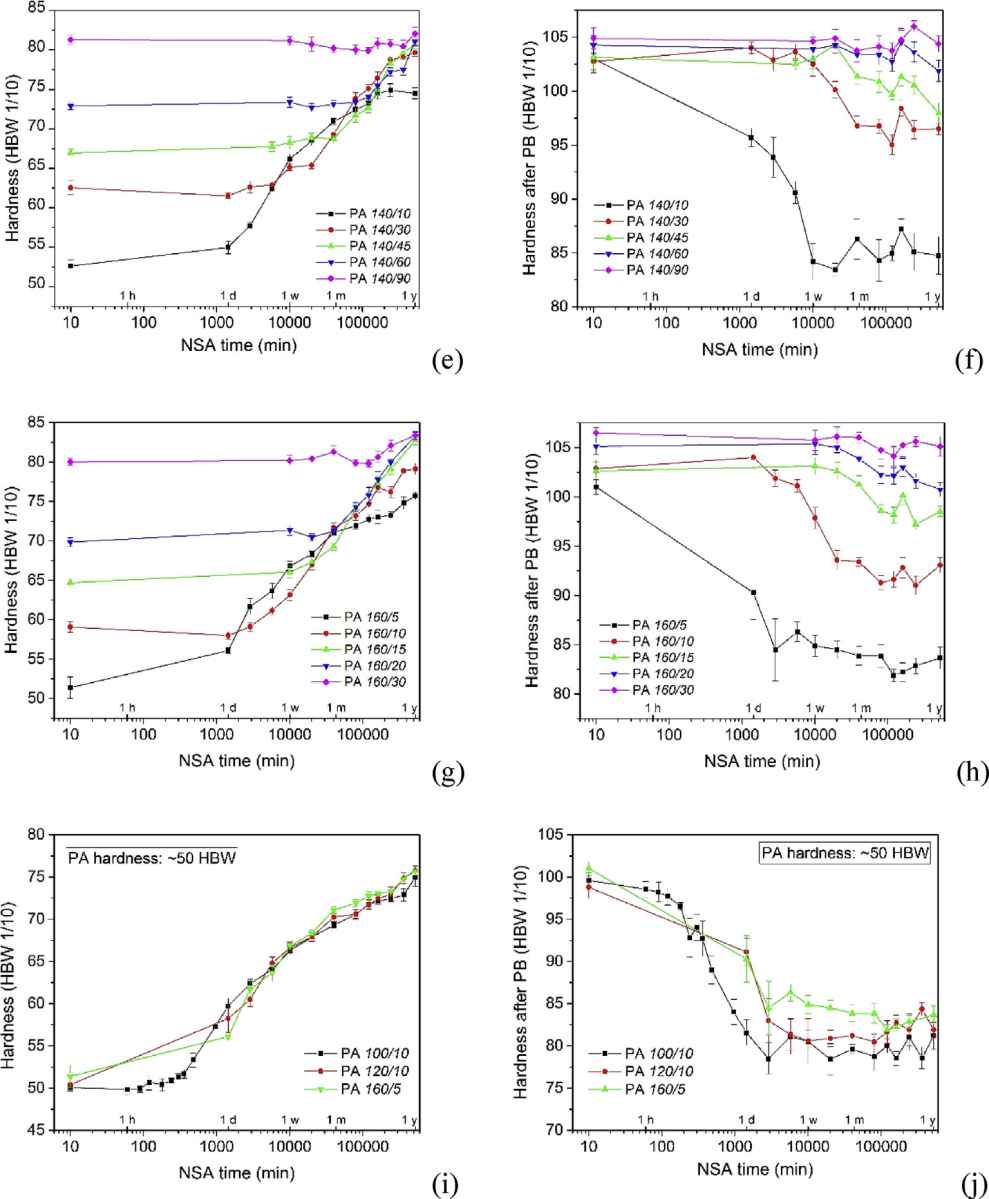

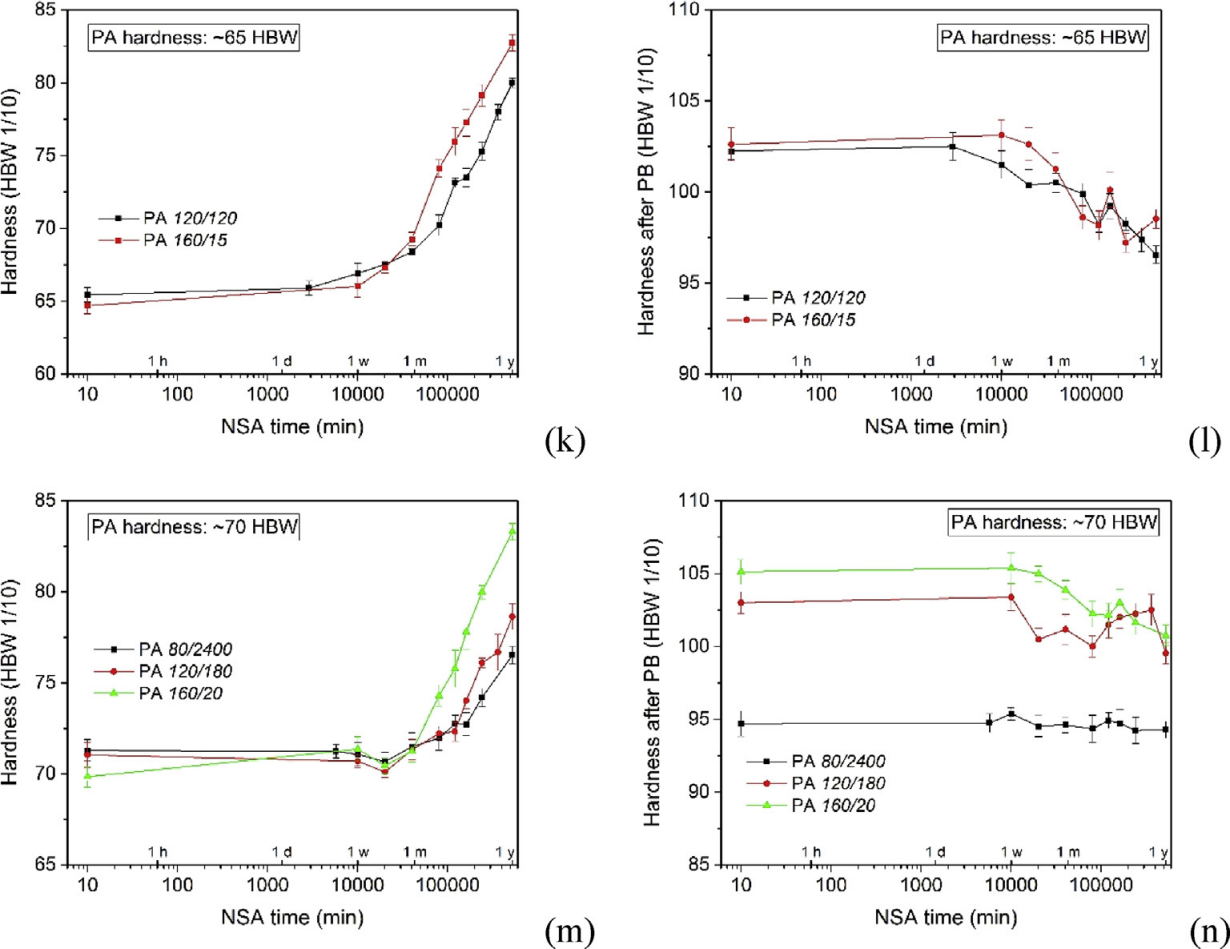
(a, c, e, g) Hardening curves during NSA after PA at various temperatures for various times. (b, d, f, h) Hardnesses after additional PB. (i, k, m) Comparison of NSA after PA to other hardnesses than those given in Fig. 3(c) of Ref. [1] (60 HBW). (j, l, n) Hardnesses after additional PB.

**Fig. 3:**
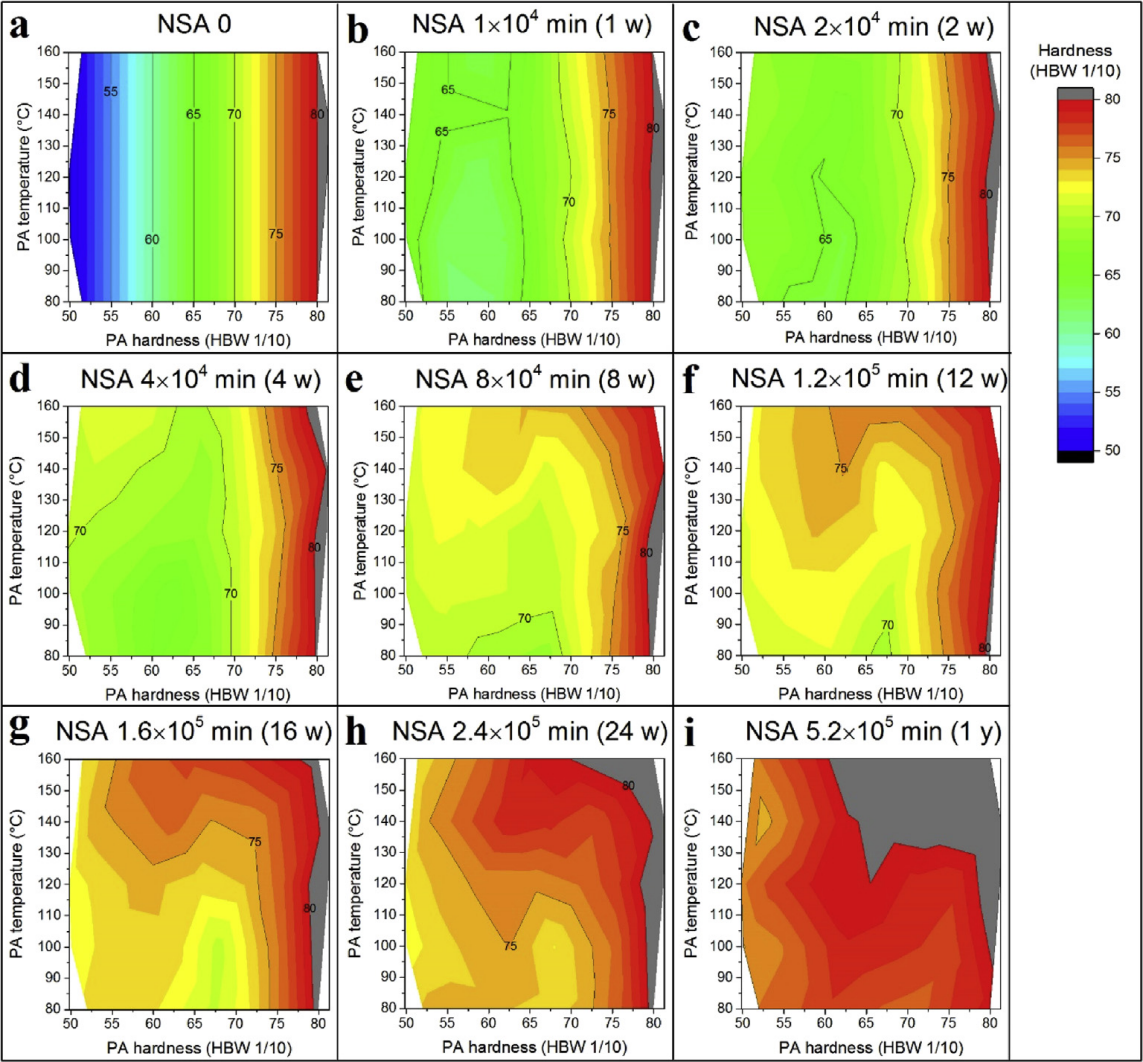
Hardness after NSA as a function of PA hardness and temperature. Different graphs correspond to different NSA times. f) is identical to Fig. 3d in [1].

**Fig. 4:**
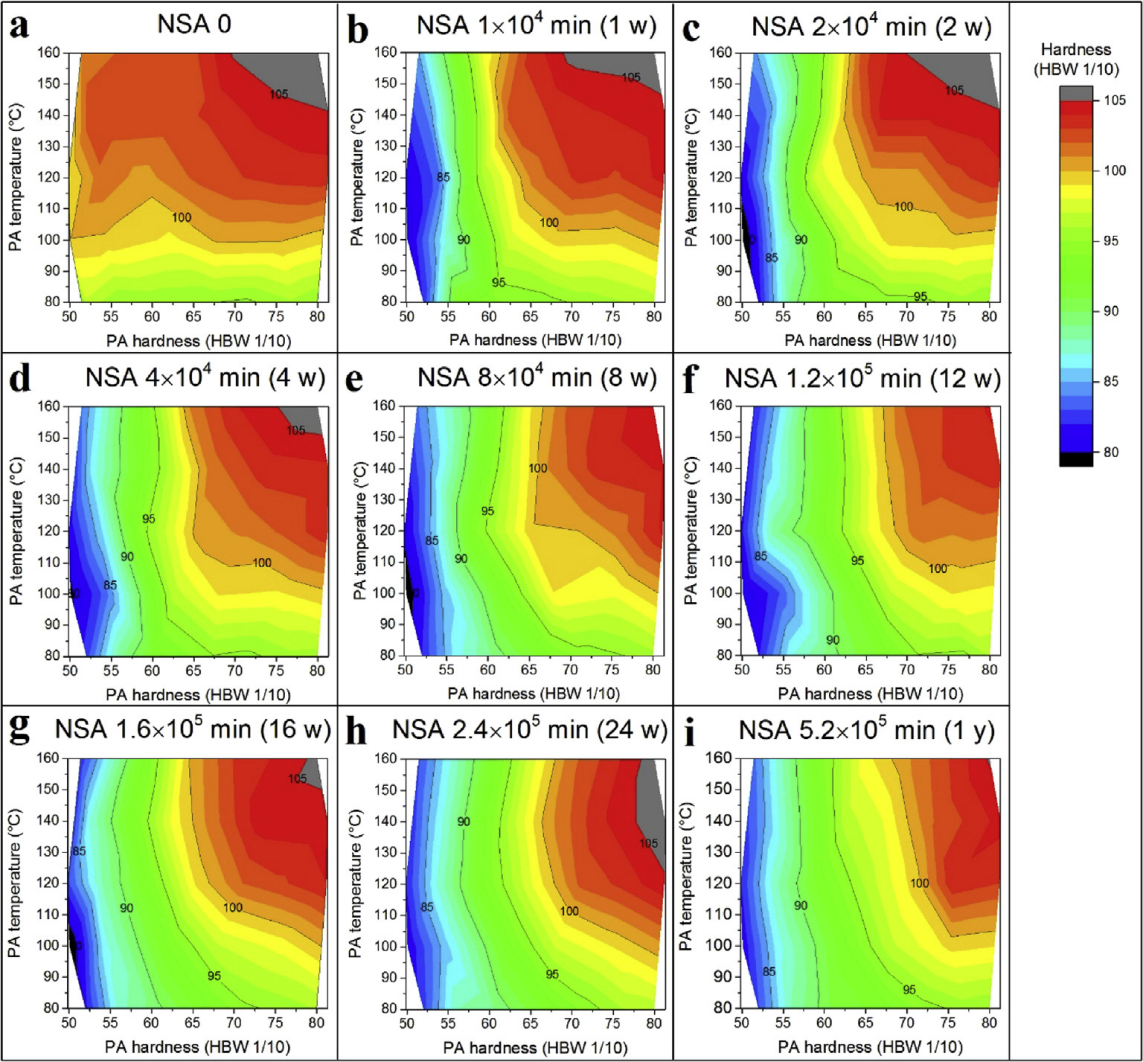
Similar to Fig. 3 but with additional PB. f) is identical to Fig. 3h in [1].

Fig. 2, Fig. 3, Fig. 4 show the hardening of the alloy during NSA and after subsequent PB for alloys that have been pre-aged under different conditions.

The curves in Fig. 2a–h are analogous to those in Fig. 3a, e of Ref. [1]), just that PA conditions differ. Fig. 2i–n correspond to Fig. 3c, g of Ref. [1].

Fig. 3 displays hardness after PA and NSA as a function of PA hardness and PA temperature, A continuous evolution from short NSA time to long NSA time is seen. NSA hardness increases first in the low PA hardness regime and at high PA temperature regime.

Fig. 4 corresponds to Fig. 3, just that a final PB was carried out. The final PB hardness changes most after the first week of NSA in the low PA hardness regime.

**Fig. 5:**
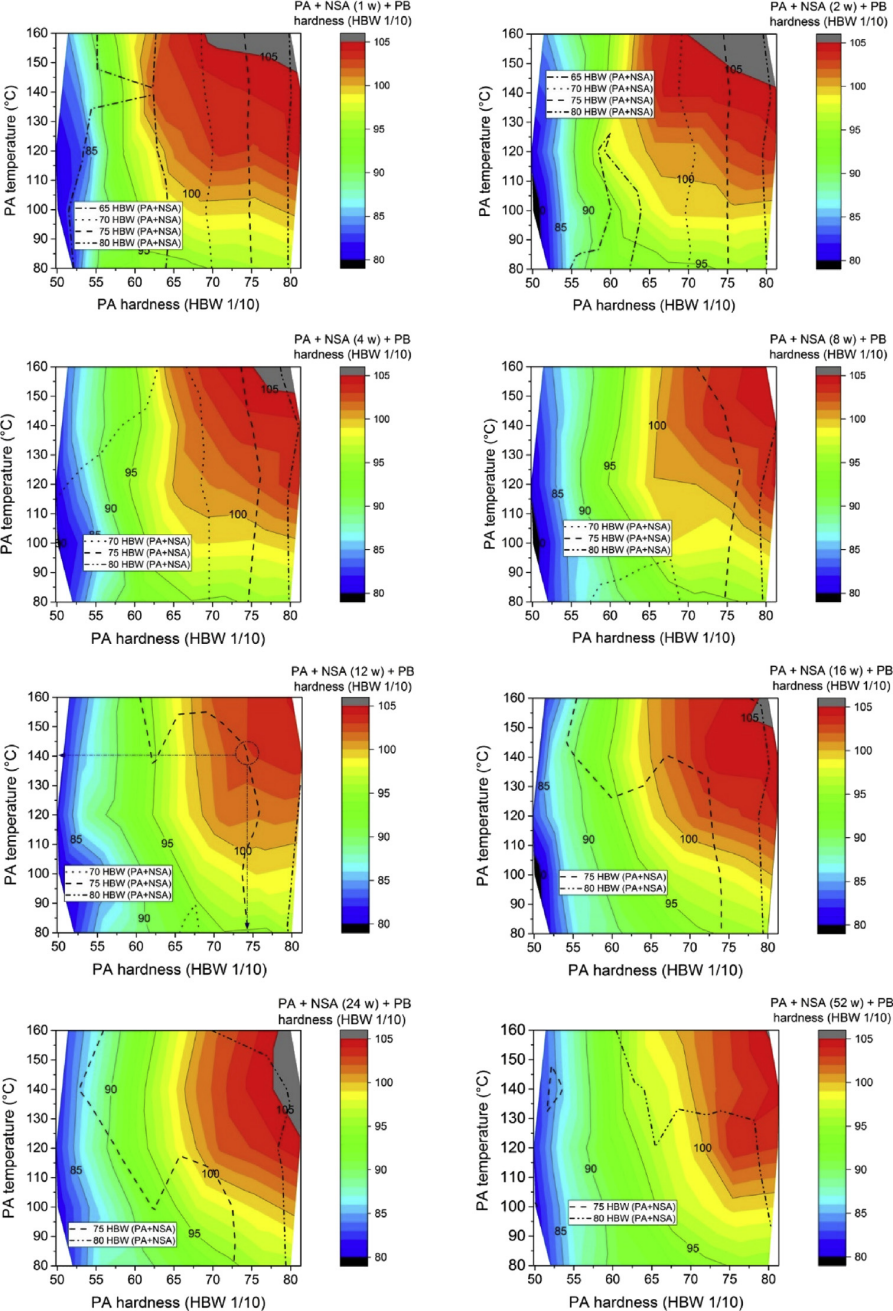
Overlay of iso-hardness lines shown in Fig. 3 over data in Fig. 4 for 8 different NSA times as given above the colour scale bar and different PA+NSA hardness levels as given in each legend. The curve for 12 weeks of NSA and 75 HBW is identical to Fig. 9 in Ref. [1].

Fig. 5 shows the overlay of iso-hardness curves after NSA (Fig. 3) on hardness plots after further PB (Fig. 4) for various NSA times. These plots can be used to determine the optimal PA strategy as described in Ref. [1].

### 2.1 Fit of hardness data by various kinetic models

We attempted to obtain kinetic parameters by fitting the hardness data during NSA, H(t), by two known functions. We present them here although none of these attempts yielded consistent kinetic parameters to illustrate the difficulties involved. The problems encountered are:

• The function does not represent the data well.
• Representation is good but the use of too many parameters makes usage of function questionable.

**Table:** 

	Function	parameters	function H(t)=
1	Single Avrami [2]	$H_{0}$, $H_{1}$, k, n	$H_{0}+\left(H_{1}-H_{0}\right)\left[1-e^{{-\left(kt\right)}^{n}}\right]$

2	Starink-Zahra [3]	$H_{0}$, $H_{1}$, k, n, η	$H_{0}+\left(H_{1}-H_{0}\right)\left\{1-{\left[\frac{{\left(kt\right)}^{n}}{\eta }+1\right]}^{-\eta }\right\}$

**Fig. 6:**
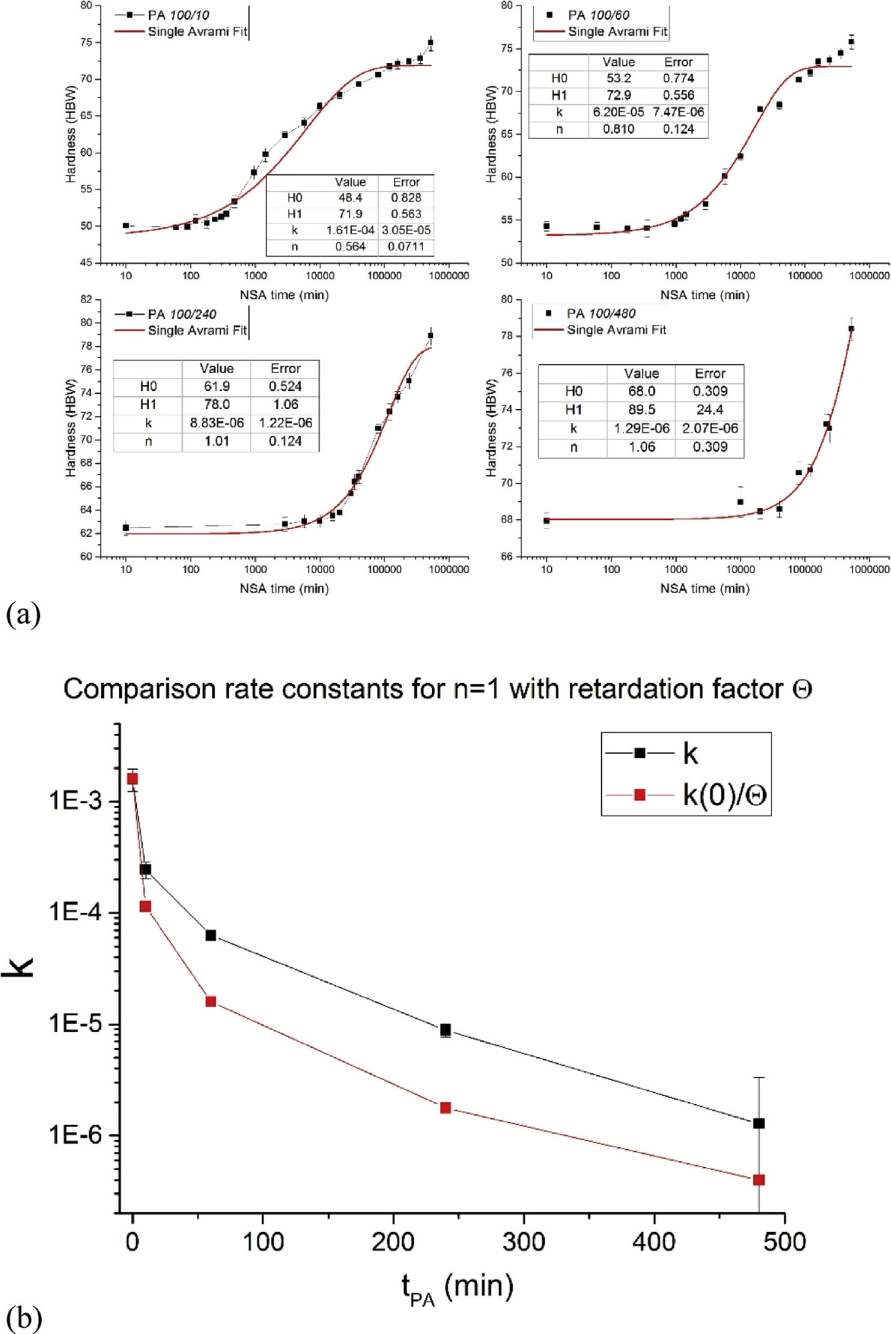
(a) Fit with Function #1. (b) comparison of rate constant k obtained by fitting with constant Avrami index n=1 and retardation factor obtained in [1].

Function #1 does not yield satisfactory fit results and the values for the Avrami-coefficient n are unrealistically low in some cases (n=0.5 for 10 min NA), see Fig. 6a. For longer PA times n≈1 is found. The conditions for JMAK are not fulfilled in our case because the vacancy fraction is continuously decreasing, which is why use of the JMAK model is questionable.

**Fig. 7:**
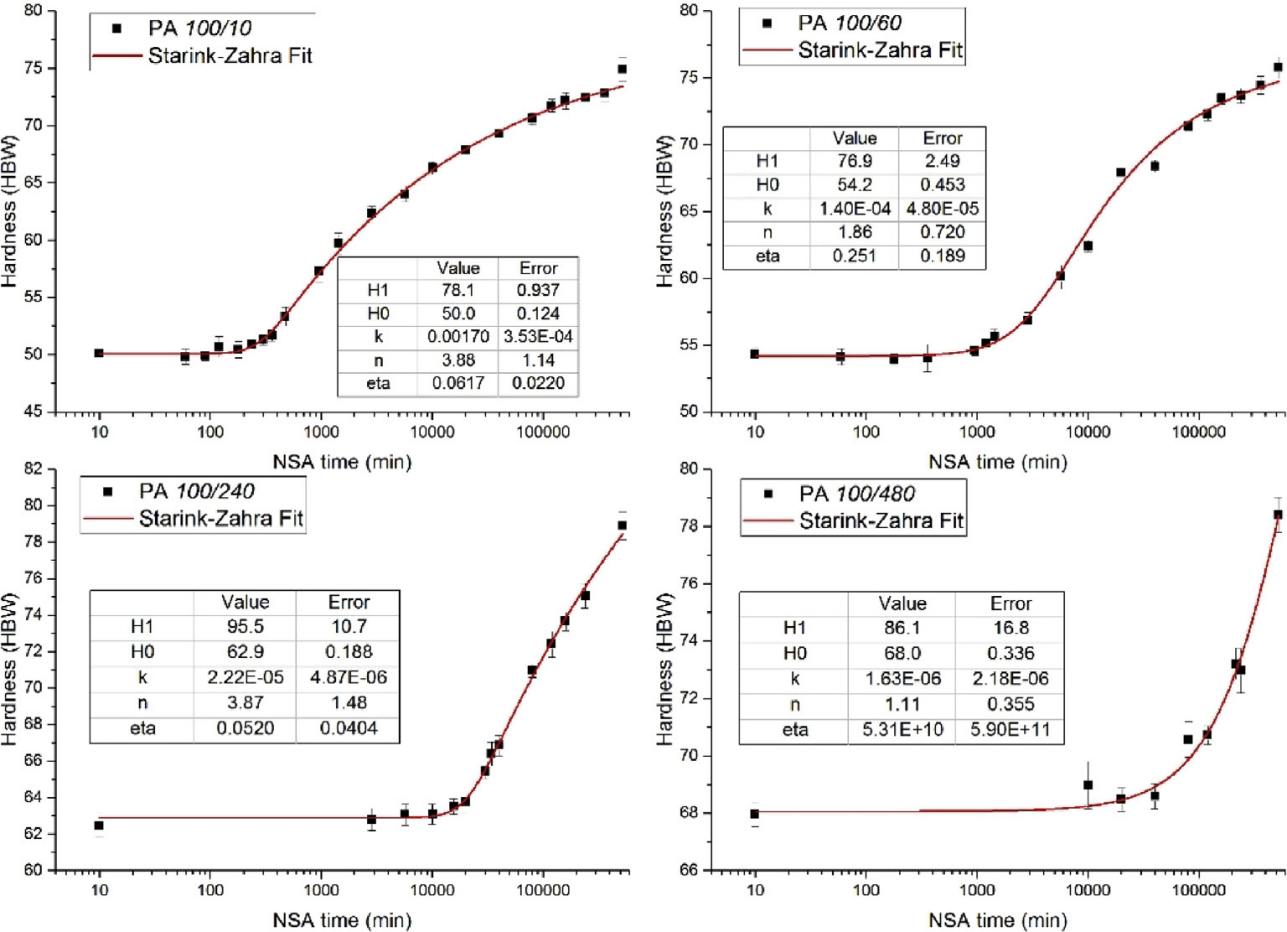
Fitting using Function #2.

By enforcing n=1 for all the fits we obtain a rate constantk that can be compared to the retardation factor ${\text{Θ}}^{-1}$ (Fig. 7 and Fig 8 in [1]). We see that the general course is the same but k tends to be larger than ${\text{Θ}}^{-1}$ by a factor up to 5.

Fig. 7 shows that function #2 fits the NSA curves quite well. However, the parameters obtained do not vary in a continuous way, indicating over-determination of the function (too many parameters).

The same applies to a double-stage JMAK function, i.e. a generalisation of Function #1 with two k and n parameters and a weight factor for the two contributions, leading to 7 free parameters. This allows for a good fit but not to derive meaningful parameters (fit not shown).
